# A system of real-time neural recording and stimulation and its potential application in blood pressure modulation

**DOI:** 10.3389/fmedt.2022.941686

**Published:** 2022-08-10

**Authors:** Anruo Shen, Runhuan Li, Yiran Li, Jinyao Guo, Jiguang Wang, Xiaohong Sui

**Affiliations:** ^1^School of Biomedical Engineering, Shanghai Jiao Tong University, Shanghai, China; ^2^School of Medicine, Tsinghua University, Beijing, China; ^3^Shanghai Institute of Hypertension, Ruijin Hospital Affiliated to Shanghai Jiao Tong University School of Medicine, Shanghai, China

**Keywords:** electrical neuromodulation, closed-loop, vagus nerve, bioelectronics, hypertension

## Abstract

Hypertension is one of the most prevalent chronic diseases that affects more than 20% of the adult population worldwide, but fortunately, most of their blood pressure can be effectively controlled *via* drug treatment. However, there still remains 5–30% of patients clinically who do not respond well to conventional medication, while the non-drug treatments currently existing are struggling with major drawbacks like irreversible nerve damage, huge side effects, and even non-effectiveness. In this study, based on the physiological regulation mechanism of blood pressure and state-of-the-art neuromodulation technique, we worked along with the vagus nerve stimulation scheme, developed, and explored whether and how a real-time neural recording and stimulation system could provide an insight into self-adaptive modulation in the blood pressure, in the hope to crack a crevice in the closed-loop treatment for resistant hypertension. Unlike traditional neuromodulation devices, additional signal recording and real-time wireless transmission functions are added to the same device to realize the features of a dynamic monitor and modulator. The system is tested both *in vitro* and *in vivo*, showing decent electrical performance of 8 kHz sampling rate and flexible stimulation outputs which sufficiently covers our needs in manipulating neural activities of interest. A relatively stable drop in the blood pressure resulting from stimulation was observed and specific patterns in the vagus nerve signals relating to blood pressure could also be primarily identified. This laid a solid foundation for further studies on the final realization of closed-loop automatic adjustment for resistive hypertension treatment.

## Introduction

Hypertension, which is defined as systolic blood pressure (BP) ≥ 140 mmHg or diastolic blood pressure ≥ 90 mmHg, is a major risk factor for cardiovascular diseases (1). According to global statistics, the number of hypertensive patients worldwide in 2020 has reached about 1.39 billion (2). With the prevalence of high-salt diets and an aging population in recent years, it is foreseeable that this number will continue to increase. Generally, the impact of hypertension takes effect slowly and is thus easy to be overlooked, but this disease should, on the contrary, be seriously addressed because of the fact of its ranking the first in the fatality rate of cardiovascular disease and its unneglectable large patient population. Antihypertensive medications, for example, diuretics, angiotensin II type 1 receptor blocker, and beta-adrenergic blocking agents, are the most universal treatment for hypertension (3). However, nearly one-third of hypertension patients still suffer uncontrolled blood pressure after taking three or more reasonable doses of antihypertensive medicine (which contains diuretics). This group of people is therefore clinically considered to have resistant hypertension (RH), a type of intractable blood pressure disorder. Although it is hard to distinctly rule out pseudo RH symptoms caused by the white coat effect or unhealthy diet, there is still an understatement that about 10 % of hypertension patients are truly suffering from RH due to poor medication adherence or physical resistance (4). For this reason, developing effective non-pharmacological treatments for RH is essential and valuable.

Physiologically speaking, blood pressure is mainly controlled by cardiac output and peripheral vascular resistance, but the regulatory mechanism is complex and mostly unclear (5). This led to a wide variety of non-pharmacological therapy attempts to treat hypertension. Among them, pure surgical methods, notably central iliac arteriovenous anastomosis, created a new low-resistance venous segment in the periphery and did achieve the purpose of lowering blood pressure (6), but this operation mode seriously damages the cardiac diastolic function and often ends up in heart failure (7). Other methods, including renal denervation and carotid body ablation, aiming to affect the activity of the autonomic nervous system by ablating the target site, also have their inescapable downsides. Renal nerves in human bodies all have their important functions to perform: Afferent nerves form many baroreceptors and chemoreceptors for sensing changes in blood pressure, and renal efferent nerves are responsible for mediating periglomerular cells to secrete renin and thereby influence the reabsorption of water and salt (8–10). However, renal denervation would irreversibly damage these nerves and even though it showed some exciting results in its second-generation trials, such as SPYRAL HTN-OFF MED, the regrowth of nerves and the possible long-term risks of irreversible damage, cannot be ignored (11–13), whereas carotid body ablation destructs the carotid body and shows only a humble short-term antihypertension effect (14). Besides, both surgeries bear a huge risk of in and post-surgery complications.

Compared with surgical methods, neuromodulation based on devices seems to have more advantages.

Related systems generally contain an implanted pulse generator and a lead that was placed at the target position. It can directly act on the autonomic nervous system through the released stimulation current and exert influence on cardiac output and peripheral vascular resistance. Furthermore, it does not damage nerves, and the stimulation effect can be adjusted by altering the parameters rapidly according to patients' current medical condition, so it possesses greater potential in the long-term treatment of hypertension. There are already many existing therapies that utilize neuromodulation, including baroreflex activation therapy, cardiac neuromodulation therapy, and vagus nerve stimulation. Baroreflex activation therapy acts on the carotid sinus, affecting the baroreflex afferents associated with blood pressure. Its antihypertensive effect has also been preliminarily verified by experiments, and an FDA-approved commercial system “Barostim Neo” has been developed based on this mechanism (15, 16). The structure of the device used in cardiac neuromodulation therapy is similar to that of a dual-chamber pacemaker. It reduces atrioventricular intervals and affects cardiac preload, which in turn decreases cardiac output. This method shows a good therapeutic effect for isolated systolic hypertension which is usually unsolved by other therapy (17, 18). The vagus nerve is the tenth pair of cranial nerves in the human body, acting as a vital part of the autonomic nervous system. It contains many nerve fibers related to heart activity and is thus believed to contain information related to blood pressure, and these signals will change synchronously with the change in blood pressure (19). Vagus nerve stimulation focuses on the parasympathetic nervous system, influencing the blood pressure-related signals ascended through the vagus nerve and related descending pathways, such as the “cardiac-vagal pathway” by electrical stimulation (20). In summary, the application of neuroregulation in blood pressure regulation is very extensive.

Looking at the neuromodulation development, there is always a call for ultra-low-power, ultra-small size, and high-precision pulse generator to better serve as an implant. On the contrary, it is also worthwhile to mention that most of the existing systems applied in current therapies are open-loop, meaning that they would only distribute stimulation with a fixed pattern pre-set during the operation. However, since blood pressure changes constantly, open-loop systems are not capable to follow this dynamic feature and provide timely optimal strategies for patients. Invalid or excessive stimulation might compromise the therapeutic effect and even trigger complications. Ideally, we hope that the system can get feedback through certain markers and correspondingly adjust the parameters to make the stimulator work in the optimal state at all times. Such a scheme could in the meantime reduce the load of patients and doctors. This is a “closed-loop” neuromodulation system. A closed-loop control system would directly detect abnormal neural signals and accordingly adjust the parameters of electrical stimulation in real-time, achieving more effective clinical treatment effects than the open-loop one. The feedback part acts a critical role in the closed-loop system. To enable the system to evaluate and set up the most appropriate electrical stimulation parameters, the feedback mechanism needs to process and analyze the characteristics of collected signals simultaneously. Therefore, such systems need to have real-time signal recording and stimulation functions at the same time.

In this study, on basis of that, we designed and developed an integrated miniature low-power system, including both signal recording and electrical stimulation functions, along with a supporting mobile phone application. The system was then applied to vagus nerve stimulation and preliminarily verified its effectiveness. Preliminary algorithms are designed to extract blood pressure-related CNAPs from vagal electroneurogram and have received promising results. It has the potential to be the closed-loop system control algorithm after further optimization.

## Materials and methods

### Overall system implementation

**Figure 1:**
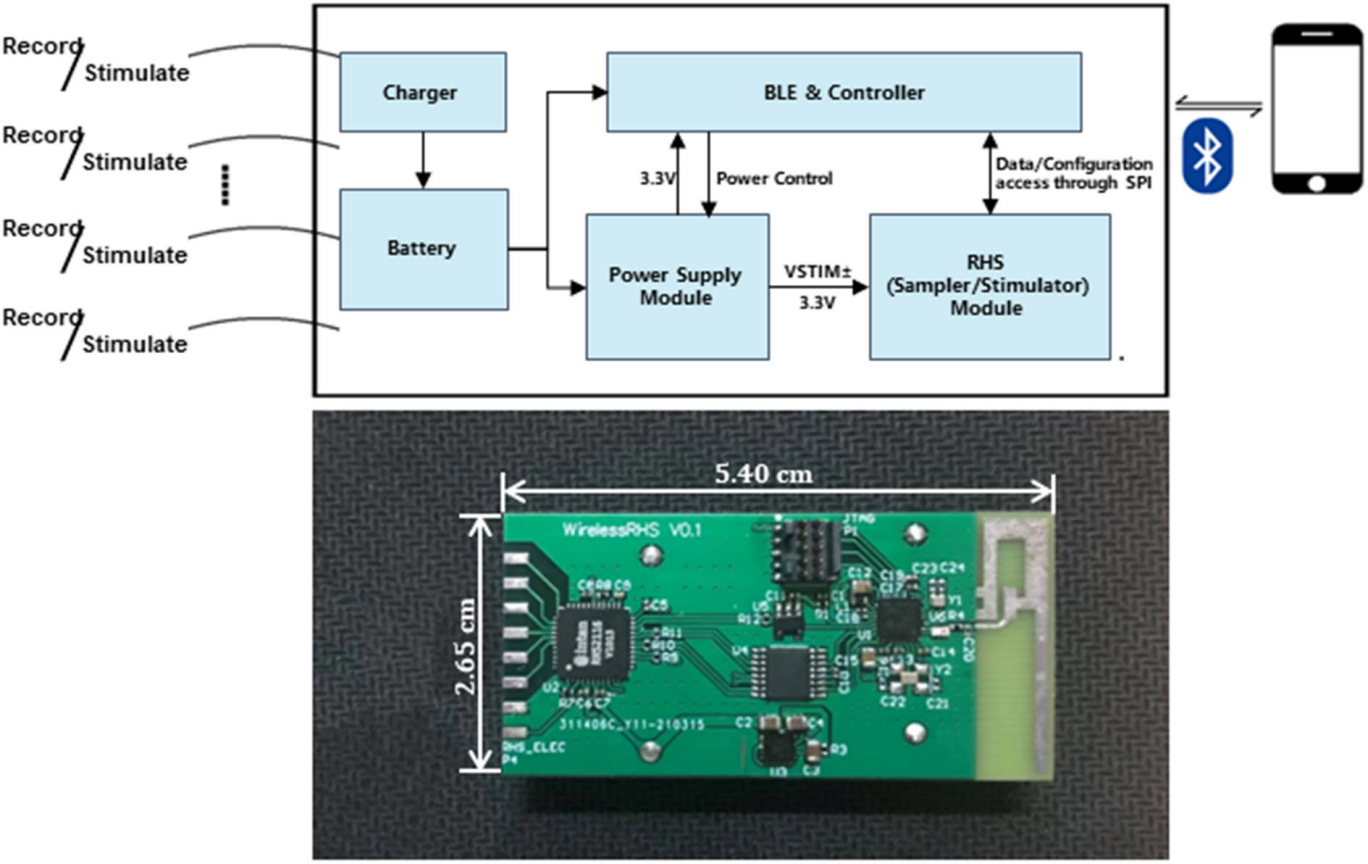
Implant device system architecture and block diagram of the IC and diagram of the developed system.

The device (Figure 1) in our design comprises sampling/stimulation multiplex electrodes and an IC, both integrated into a cm-sized package and communicating with an external program. Major blocks in the IC include a power supply module, controller module, and a programmable sampler and stimulator.

The power supply module is responsible for powering the whole system. It is provided by ±6 V external batteries, from which the circuit generates a regulated DC supply rail (V_LDO_ = 3.3 V) for powering other modules, and determines the maximum value of the stimulus current amplitude.

The controller module acts as a command center and coordinates different tasks. In our case, software coding is used to simulate serial port communication with the other chip to utilize the sample and stimulate functions. Meanwhile, it also interacts with App through the BLE service. This system allows full configurability of the stimulation pattern which is useful for rapidly adapting stimulation during closed-loop operation.

The system records real-time neural electrical signals from the vagus nerve and outputs electrical stimulation based on pre-set customized parameters. The stimulus is performed on the vagus nerve, neurons of which would consequently affect related sympathetic transmission through a set of neurological pathways to achieve the purpose of lowering abnormal blood pressure.

The wireless link is also a critical part of implantable devices. To realize the control of recording and stimulation, the implanted system is wirelessly connected to the external system through Bluetooth 5.0 in our design. It is mainly responsible for the interactive control between the inner and outer body systems. To be specific, the external system could receive the real-time data transmitted from the implantable system and also send instructive commands to the system.

The implantable system works with an external system: a customized mobile App. It is a family-side controller for the device's functionality. From the App, the user could select to utilize the specific function: Recording or Stimulation. It also has auxiliary functions, such as Bluetooth pairing and signal visualization (more to be elaborated on in the coming section).

### Signal recording

The electrical signal acquisition function is realized using the RHS2116 chip from Intan Technologies of the United States. This is a bidirectional electrophysiological interface chip especially developed for the field of neuroscience. In our design, the communication process of the serial ports is simulated by software to further optimize time sequence arrangement. Four I/O ports are selected for customization, respectively, simulating CS, SCLK, MOSI, and MISO channels, strictly following the timing diagram relationship and constraints in the datasheet (t_SCLK_>40 ns, t_SCLKH_ = t_SCLKL_>20 ns, t_CS1_ = t_CS2_>20 ns, t_MISO_ <12 ns, t_CYCLE_>1,400 ns).

To further analyze the data collected by the hardware system and find the components related to hypertension in the vagus nerve electrical signal, data storage in the software part is essential. Due to the large amount of data that needs to be stored, it is not expected that the data and the App have a symbiotic relationship for huge amount of data, which will inevitably overload the App and consequently interferes the reaction efficiency. Thus, the data are stored in the external memory assigned to the software at local memory. The MCU in the system wirelessly transmits the data to the mobile App *via* low-energy Bluetooth. The external Bluetooth device, that is the mobile phone, received data packets and displayed them in the form of a waveform for real-time monitoring. By choosing the Export button designed in the App, the real value of neural signals could be saved locally for further analysis. The recording function can also be stopped by pressing Disconnect button in the App.

### Electrical stimulation

According to the datasheet of the RHS2116, each stimulation amplifier module contains a constant current stimulator capable of driving a positive current between 10 nA and 2.55 mA or the negative current amplitude determined by a specific register configuration. In this design, this function is implemented by selecting the stimulation channel, setting the stimulation parameters (amplitude, pulse width, and polarity), turning on the enable, and turning off the enable to execute the output of a single electrical stimulation. Multiple channel stimulation could be realized at the cost of stimulating frequency. The stimulation command is placed in an infinite loop in the logic to realize the function of continuous electrical stimulation.

Given the feature of the electrical nerve signal, the stimulating waveform is ideally expected to be a spike. However, in a practical situation, we use the negative first and then the positive rectangular wave to mimic the spike. The bandwidth of each could be changed to accommodate different situations. In our study, the symmetrical biphasic voltage pulse was generated by the current-source stimulator with balanced cathodic and anodic phases and a load resistance which convert the current pulse to voltage pulse. The stimulation amplitude range was set at 10nA−2.55mA with a minimum 10 nA resolution. The frequency that the system can output ranges from 0 to 6.7 kHz, which is adequate to suppress neural excitement so as to downregulate blood pressure for our study.

### Wireless communication

Considering the particularity of implantable neuromodulation system, the transmission of data to the outside of the body needs to be realized by means of wireless communication technology. Through the investigation, it is found that the custom wireless protocol is difficult to realize, so this design chooses to take advantage of the existing mature protocol for communication, such as WIFI, Bluetooth protocol, and Zigbee.

As shown in Table 1, we took into account the data throughput requirement of the communication rate, power consumption, and data safety factors and decide to adopt the Bluetooth 5.0 protocol as the method of wireless communication between the implanted closed-loop control circuit and the host computer. Following this, we selected the MCU that supports low-energy Bluetooth as slave and mobile phone application as master for user convenience considerations. Common mobile operating systems are iOS and Android. According to their respective protocol, the Bluetooth connection interval of iOS is 20 ms, during which information could be exchanged up to 4 times, and the maximum communication rate is 6 KBytes/S; while the connection interval of Android is 7.5 ms, and frequency of interaction can be up to 6 times per interval, the communication rate is 16 KBytes/S. The real-time nature of this design requires that the faster the data transmission rate, the better. Therefore, we chose Android software for development.

**Table 1:** Comparison of parameters of different wireless communication technologies (24).

**Name**	**WiFi**	**Bluetooth**	**Zigbee**	**RFID**	**NFC**
Maximum transfer rate	11 Mbps	1 Mbps	250 kbps	1 kbps	424 kbps
Communication distance	100 m	10 m	10–75 m	1 m	20 cm
Frequency band	2.4 GHz	2.4 GHz	2.4 GHz	2.4 GHz	13.56 MHz
Safety	Low	High	Medium		Ext. High
Anti-interference			Low		
Power consumption	10–50 mA	20 mA	Ext. Low	10 mA	10 mA
Application	Internet, PC, PDA	Communication, automotive, IT, multimedia, industrial, medical, education, etc.	Industrial control field, Internet of Things	Read data, replace bar codes, access control	Cell phones, near field communication, smart cards

It is hard-coded in the program that the implant and the mobile App are a one-to-one registered connection out of safety concerns. The connection established not only transmits collected electrical signal values but also provides basic human–computer interaction to control specific functions. When the stimulus/collection button is pressed, the MCU wirelessly receives control commands and stimulation parameters from the mobile App. Commands transmitted through Bluetooth will be accepted by the hardware system, and the corresponding function will be immediately executed. Between the updates of stimulation parameters, the implantable device is worked without intervention from the outside as a standalone system.

### Other considerations

The range of considerations affecting device performance is wide and varied. It concludes size, packaging schemes, system stability, robustness, and battery endurance. From an engineering perspective, as a prototype for a future closed-loop system, our consideration at this stage primarily focuses on the realization of real-time neural signal collecting and nerve stimulation function. However, a few critical electrical properties and human-oriented concepts are pondered and infused into the device prototyping process. In the following sections, we will address the topics which relate most directly to our main focus.

#### Noise level

The empirical value of vagus nerve is around tens to hundreds of microvolts, which is very faint compared to other forms of the signal. In other words, this feature of vagus neural signal is setting an extremely high barrier for the sensitivity level of sampling tools. To reduce any possible interference generated by the circuit itself, the system used as few components as possible, integrating most analog signal processing into the MCU. At the same time, LDO regulators are used to derive lower output voltages from battery instead of DC/DC switch because LDO can provide a cleaner power to the whole system which is more suitable for noise-sensitive applications. In terms of determining the communication mode between the RHS2116 and MCU, low-voltage differential signaling (LVDS) is adopted because due to its double-ended driving principle and benefiting from its characteristics of differential input and low-voltage swing, has stronger anti-noise capability. We also take it into consideration when designing the PCB. A four-layer structure is adopted with the ground layer and power layer protected in between two separate signal layers. The layout design follows principles of minimizing traces and through-board vias, as well as the rules of line width and line spacing to avoid electromagnetic interferences on the board.

However, the noise level as a whole in real practice is difficult to be numericized. In general, all samples have similar noise levels, yet the specific value still varies from time to time. We conducted a test short-circuiting the sampling electrode with GND and detected a random thorn of 5 μV, the value of which is acceptable compared to the signal value we are interested in. Nevertheless, data cleansing will be taken care of in the later processing algorithms to minimize the effect of this noise.

#### Power consumption and size

Power consumption is of great importance in digital systems. Keeping the consumption low increases energy security and avoids local overheating resulting from long time working. Besides, it is a crucial electrical property in our design because battery life of portable systems is directly limited by the consumption rate and as a future implant candidate, it should aim to be enduring.

Multiple considerations are made regarding hardware selection and software design. The original version of the system (the one that is used for *in vivo* validation) uses button cell batteries for power supply, which is a normal choice to cooperate with the Bluetooth Low Energy. However, later we realized that upon the powering of RHS, its instant current consumption will exceed 30 mA, and even in the steady state, there will also be a 5 mA or higher current, the consuming rate of which could barely be supported by the button batteries. So based on this issue, multiple versions of the hardware system were later prototyped to seek for a more suitable power supply, as well as continue to decrease the power consumption.

Considering the need for a reasonable volume, a single-cell rechargeable lithium battery along with a DC/DC power supply system is used and we redesigned the circuit to link the LDO input of RHS2116 VDD directly to the battery. In the aspect of software improvement, we turn off the power supply of RHS in ultra-low-power standby mode. The test result found a compromise between size and battery capacitance. Larger the battery capacitance, larger the size of the battery which dominates the final size of the overall system. Two representative version comparison could be summarized as in Table 2.

**Table 2:** Tradeoff between size and battery capacitance.

**Power Cons. (lin@3.7V, mA)**	**Est. Battery Life (Hr)**	**PCB Size (W × L × H, mm)**
32	28.1	31.5 ×60.6 ×11.5
15	24	24.7 ×55 ×10

*1 Power consumption is tested with 3.7 V input voltage, one sampling channel on, no stimulation output, and no LEDs on*.

*2 The size measured includes the RHS connector but does not include the case*.

*3 Estimated battery life is calculated by*

$Est.\;Battery\;Life=\frac{Capacitance\times 0.9}{Power\;Consumption}$

*0.9 is the factor considered the tolerance of capacitance. The real battery life may vary depending on whether the stimulation function is on or off and the number of channels enabled*.

#### User-friendly interface

We developed the mobile App in the Android system with a graphical user interface (GUI) by using the development platform Android has provided (Android Studio 4.1.2, Google, USA). The reason for choosing Android over iOS is that the former has a higher transmitting rate and thus presents a greater efficiency in the high-level real-time case.

Digitized raw data are transmitted from the implantable device to the App. At the beginning of the use, Bluetooth pairing is required. Once this connection is established, the GUI offers options of Record and Stimulate functions for the user to choose from. The GUI will display waveforms of the neural signal detected if the Record button is pressed. The waveform could automatically adjust its proportion to the screen canvas based on its actual amplitude. On the contrary, if Stimulate button is pressed, the GUI will be able to update stimulation parameters and periodically distribute certain stimuli at the same time. The customizable parameters include stimulation amplitude (10 nA−2.55 mA, 10 nA resolution), stimulation frequency (0–6.7 kHz), and stimulation duration (0.083 μs resolution).

### Signal processing algorithm

#### Data-analysis methodology framework

**Figure 2:**
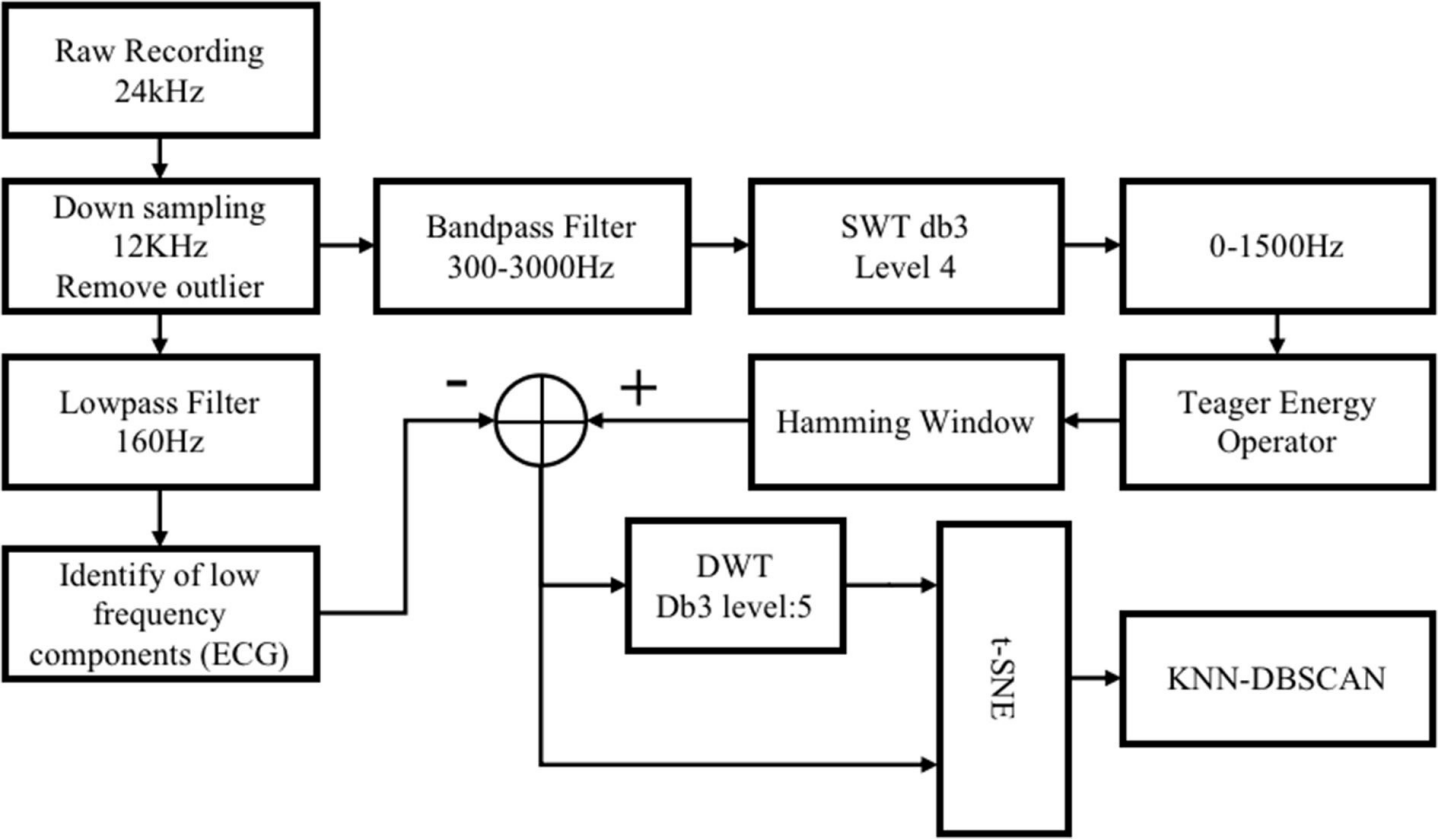
Schematic diagram of the compound neural action potential (CNAP) detection and the clustering algorithm.

To achieve closed-loop regulation of blood pressure, appropriate indicators are needed to prompt the timing of regulation and the selection of parameters. To begin with, the identification of blood pressure-related components in the vagus nerve electroneurogram (VENG) is essential. Figure 2 is the schematic diagram of the compound neural action potential (CNAP) detection and the clustering algorithms with steps carried out to extract neural responses. All the processing is implemented within the software “MATLAB” on the computer.

At the first stage of algorithm development, we adopted the VENG commercial signal recording system “TDT System 3” with neurophysiology module “RX7” as data acquisition equipment. The sampling frequency of the TDT system is 24,414Hz which is more than sufficient to collect neural signals for the sequential analysis. As an already mature commercial product, the kit is providing a more stable and ample data source for researchers to solely focus on mining the potential correlations. Therefore, algorithms were developed on the data collected by TDT system and were later applied and further cross-examined on the electronic system designed in this work. The experimental paradigm of data collection is the same as the one introduced in Section Pre-processing.

#### Pre-processing

Raw recording data are downsampled to 12 kHz, which reduces the load of calculation and retains valid information under the 6 kHz Nyquist frequency. Extremely large empirical thresholds are applied to remove significant non-neural interference, such as some abiotic interference and excessive EMG (usually >150 mV).

#### CNAP detection

##### CNAP pre-detection

Generally, the raw data still contain a lot of useless and confusing information despite of efforts made in hardware design to reduce noises; thus, it is necessary to extract spikes to further purify our biosignal and unveil the relationship between neural signals and BP values.

**Figure 3:**
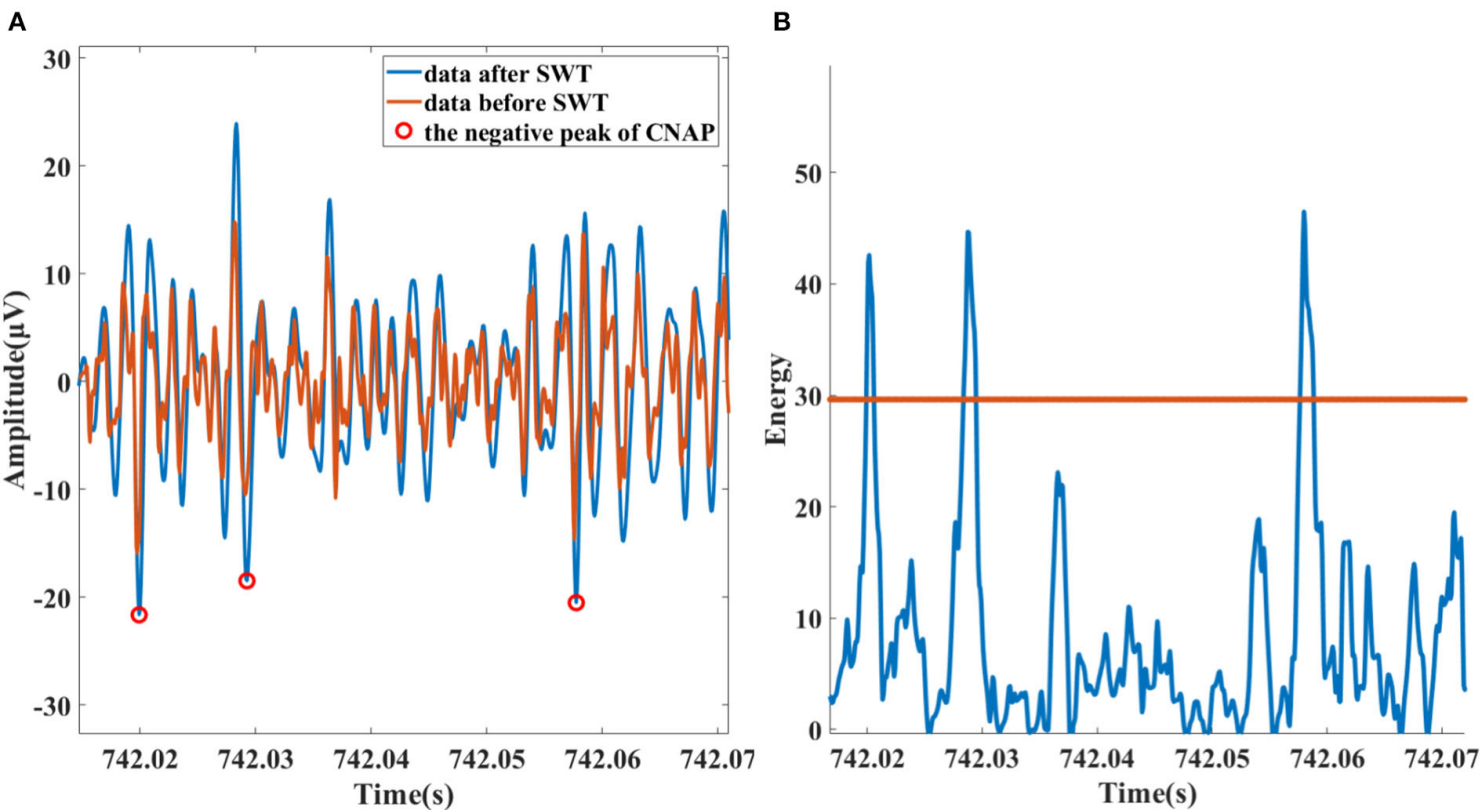
**(A)** Data and the peak of detected neural spike before (red) and after (blue) SWT enhancing. **(B)** Spike detection based on Teager energy operator.

The data are first passed through a 300–3,000 Hz band-pass filter to attenuate residual low-frequency interference components, such as ECG and EMG. The next step is to “highlight” the CNAP and eliminate some high-frequency noise interference for that most of the BP-relevant information is contained between 300 and 1,000 Hz frequency band. The stationary discrete wavelet transform (SWT) with “db3” wavelet is calculated, and then, the 0–1,500 Hz sub-band was selected. As shown in Figure 3A, spike amplitude was enhanced in 300–1,500 Hz sub-band as a result. High-frequency noise has also been noticeably smoothed.

The identification of spikes is based on the method of smoothed Teager energy operator (TEO). The first-order TEO is a non-linear energy operator:


$$\begin{array}{r} \psi \left[x\left(n\right)\right]=x^{2}\left(n\right)-x\left(n+1\right)x\left(n-1\right) \end{array}$$

It is mathematically sensitive to short-term high-frequency signals. This method is adopted because the spikes and high-frequency noise exactly satisfy this feature as it is recognized that high-frequency noise is mostly sharper while the spike lasts longer. After being smoothed with hamming window, high-frequency noise components of TEO are attenuated. Subsequently, with a suitable threshold, spikes are identified, the process illustration of which is shown in Figure 3B.

##### CNAP cleaning

Nevertheless, the quality of the signal obtained from animal experiments could be sometimes poor. Although a band-pass filter has been utilized to filter out the ECG, the residual rhythmic sharp signals from heart beating still have the possibility to be enhanced with SWT and then be mistakenly detected as CNAP. So, we need to eliminate these signals in this step.

**Figure 4:**
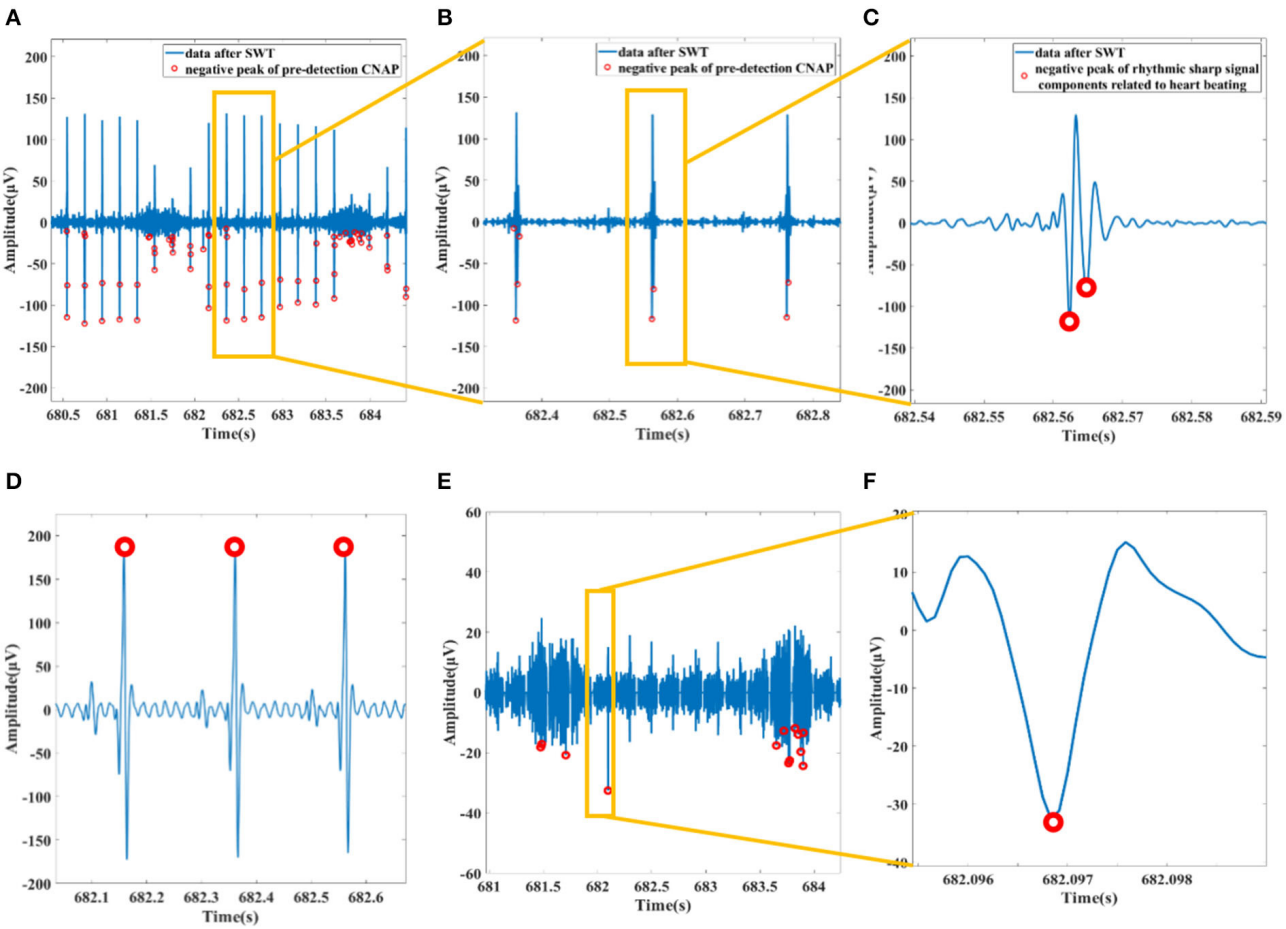
**(A)** Data after SWT and pre-detection CNAP. **(B)** Filtered ECG mistakenly detected as CNAP. **(C)** An example of a rhythmic sharp signal from heart beating. **(D)** Preprocessed data passing through a 160 Hz lowpass filter and the R wave of ECG detected. **(E)** Data with ECG and related spikes removed. **(F)** Waveform of a CNAP.

The pre-processed data pass through a 160 Hz low-pass filter to magnify the components of the ECG. Among them, the QRS wave of ECG is obvious and is located with threshold (3 times of standard deviation). On the basis of the original outcome of CNAP pre-detection, we remove all detected data segments that fall within the QRS segment to get a relatively “pure” vagal CNAP. The subtracted signal is then used for subsequent statistics of firing rate and cluster analysis. The process is shown in Figure 4.

#### Dimension reduction and clustering

After the peak point of CNAP was found, it was considered as the center of the CNAPs. A time window of 4 ms (48 data points) data segment around the center was then extracted as CNAP segment. To highlight the weight of frequency components of CNAPs on classification, a five-level DWT wavelet is applied to all CNAPs to extract its frequency information, and these wavelet coefficients are supplemented into every CNAP. Each CNAP contains 118 data points after the feature extraction.

To solve the problem of high-dimensional data sparsity and lessen the huge computational load, dimension reduction is essential. Principal component analysis (PCA) was used to reduce it to 30 dimensions, and t-distributed stochastic neighbor embedding (t-SNE) was used to further reduce it to two dimensions. Consequently, the similarity between samples was therefore represented in distance in a 2D plane. Similar samples are dense, and dissimilar samples are alienated.

As a density-based clustering method, density-based spatial clustering of applications with noise (DBSCAN) is suitable for the clustering of data samples after t-SNE. DBSCAN does not need to know the number of clusters to be classified in advance, and its classification effect depends on the choice of its two hyperparameters “Eps” and “minPts.” The selection of these parameters significantly affects the clustering effect and is generally determined based on experience. KNN-DBSCAN is a variant of DBSCAN which combines k-NearestNeighbor (KNN) to realize DBSCAN without experiencing super-parameters and obtains a robust effect (21). Given considerations to these aspects, KNN-DBSCAN was chosen in this case.

### *In vivo* testing

#### Intrafascicular carbon nanotube yarn electrodes

To cooperate with the recording and stimulation functions of the circuit, the electrodes should meet the requirement of the low impedance, high mechanical strength, and better biocompatibility. Carbon nanotubes yarn (CNTy) satisfies all of these characteristics. Compared with platinum–iridium alloy materials, its outstanding flexibility makes it less damaged after surgical placement. CNTy with a diameter of 30 μm (from Suzhou Institute of Nano-Tech and Nano-Bionics, CAS) was selected as the electrode material, the surface of which was insulated with C-Parylene (by Suzhou Kairui Nano Technology Co., Ltd.). We applied a low-pressure chemical vapor deposition method to insulate the electrode, controlling the one-side insulation thickness to be around 3–5 μm.

The electrode is designed as a “shielded wire-transition wire-CNTy” structure. The transition wire uses a stranded stainless steel wire from A-M Systems., Ltd., which consists of seven single wires with a diameter of 25.4 μm. The external layer is insulated with polytetrafluoroethylene, the diameter after the insulation is 139.7 μm, and the length is cut to 5 cm. The diameter of the shielded wire is 2 mm. The 1-cm insulating layer near the connection part of each section is peeled off and wound to connect, the conductive silver glue was applied to the connection part and dried at 150°C for 15 min. For the last step, phenolic epoxy resin was applied to the connection (22).

#### Electrophysiological experiment

**Figure 5:**
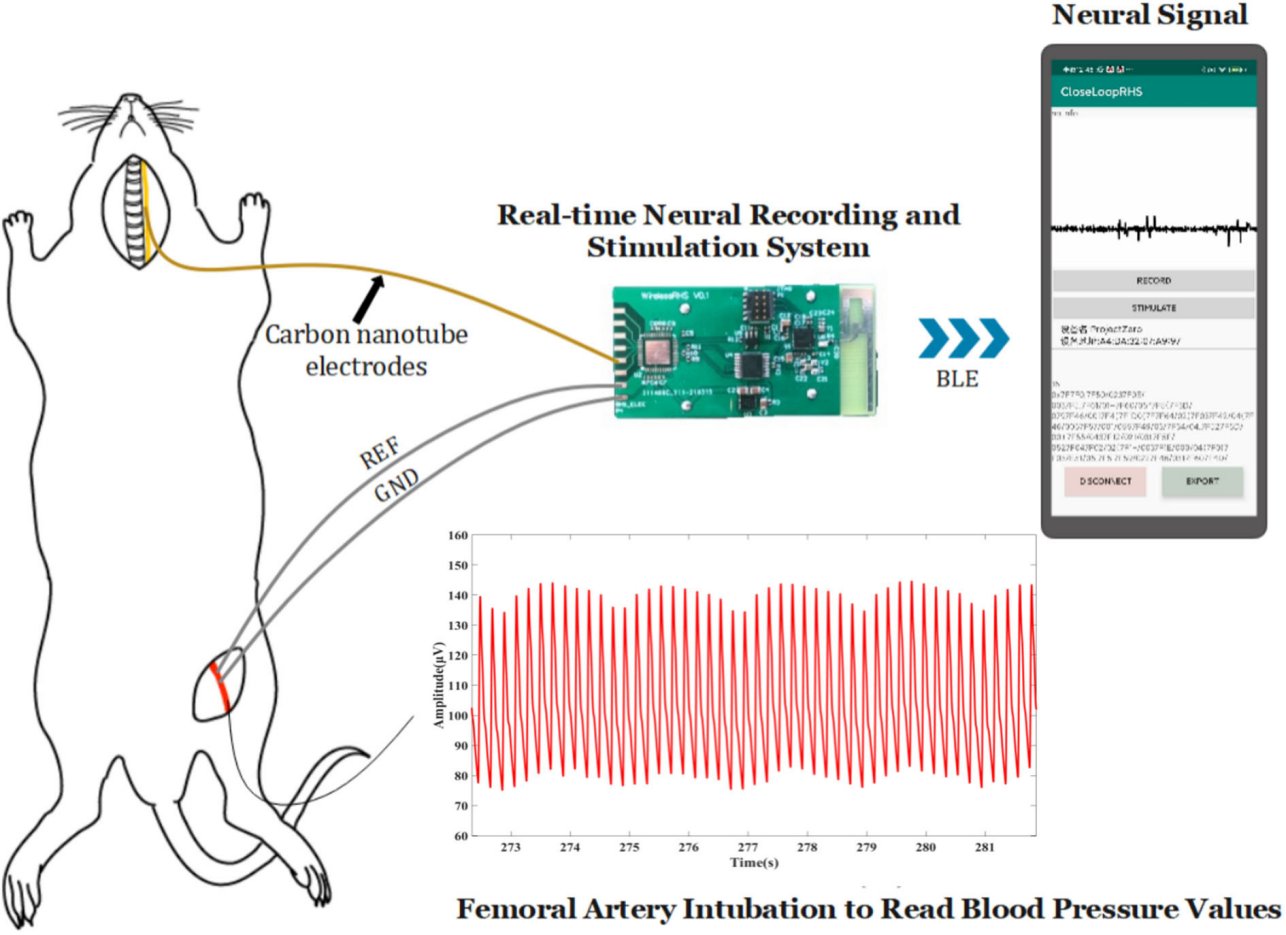
Actual study design.

Figure 5 shows the experiment diagram of the whole system in the application of blood pressure modulation on a rat. About 300–350 g Sprague-Dawley (SD) rat was selected as experimental animals. The rat was injected with 3% isoflurane gas, anesthetized for 5–10 min, and then maintained anesthesia with 1.5–2% isoflurane gas. Then, the rat was dissected to expose the left cervical vagus nerve and left femoral artery. The electrode was implanted into the vagus nerve under the guidance of a tungsten needle, and it was connected to the recording and stimulation system. The blood pressure of rats was meantime obtained by femoral artery cannulation, and the tubing was made of polytetrafluoroethylene. These data were used to testify the effect of lowering blood pressure on the stimulation function. The blood pressure recording device is the NL108A (hydraulic pressure sensor, Digitimer Company), its sensor is connected with Micro1401 (Cambridge Electronic Design Company), and the data are transmitted to the computer and analyzed with the software “spike9.” Rats with stable anesthesia were injected with phenylephrine every 10 min to achieve a periodical “sharp rise-slow fall” change of blood pressure. The data acquired from the recording function were analyzed with the software “MATLAB” on the computer (23).

## Results

### Benchtop validation

In this work, an electronic system device has been primarily built to possess the functions: (1) wireless data transmission, (2) real-time neural signal recording, (3) adjustable-pattern stimulation, and (4) algorithmic analysis to unveil the relationship between neural signal and BP value. Therefore, the work covers all the major components of the closed-loop neuromodulation protocol. Table 3 is a summary that compares the functions that an open-loop, an ideal closed-loop, and our current prototype modulating system have. In the following sections, we will exhibit the testing performance of the core functions we have realized.

**Table 3:** Functionality comparison of open-loop neuromodulation, ideal closed-loop neuromodulation, and current prototype in this study.

	**Open-loop neuromodulation**	**Ideal closed-loop neuromodulation**	**Current prototype**
Sampling	No	Yes	Yes
Stimulation	Yes	Yes	Yes
Adjustable pattern	Yes	Yes	Yes
Automatic-adjust	No	Yes	No
Data transfer	No	Yes	Yes
Real-time	/	Yes	Yes
Feedback loop	No	Yes	Not fully
Decoding	/	Yes	Yes
Feedback regulation	/	Yes	No

#### Wireless data communication

SmartRF™ Studio is a Windows application that can be used to evaluate and configure TI's low-power RF devices. This application helps designers of RF systems to easily evaluate radios early in the design process. In the test of the Bluetooth transceiver capability, we used the CC2640R2F development board to cooperate with the prototype we designed and produced, and tested the Bluetooth transceiver function of the prototype under the configuration of channel 17, 2.4 GHz RF frequency, and 5dBm transmit power. As a result, the hardware device can send and receive normally. The RSSI (Received Signal Strength Indication) parameter indicating the received signal strength during the sending and receiving test is −49dBm, which is in an acceptable range.

#### Recording

**Figure 6:**
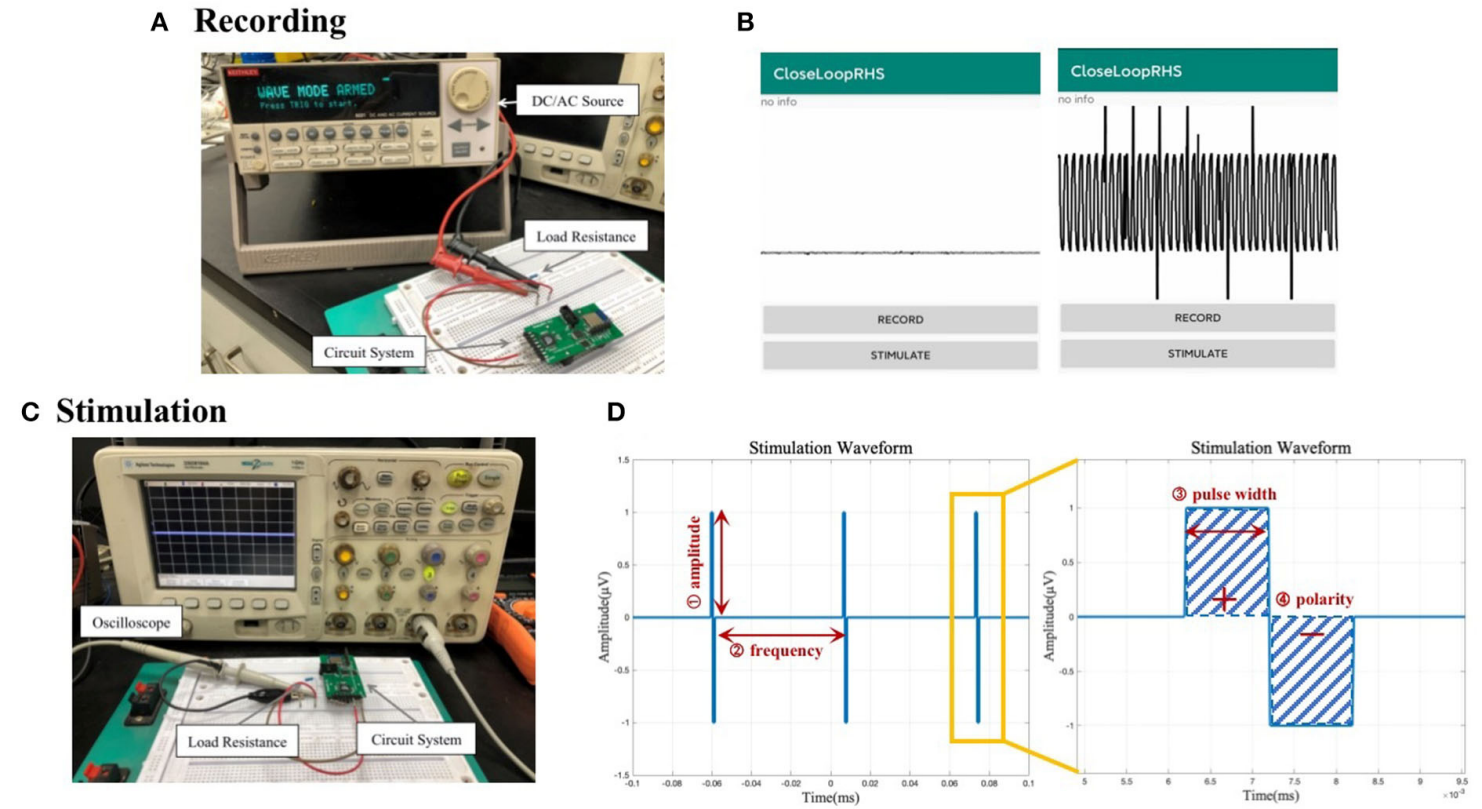
**(A)** Circuit connection diagram for the system recording functional test. **(B)** Waveform display of the data recorded by the system acquisition function on the mobile phone. **(C)** Circuit connection diagram for system stimulation functional test. **(D)** Illustration of output stimulus with adjustable parameters: amplitude, frequency, pulse width, and polarity.

The signal generator was connected with the hardware acquisition channel as shown in Figure 6A. According to the datasheet of RHS2116, the maximum amplitude of the electrical signal that can be collected by the channel is 5 mV, so this test uses a signal generator to generate a sine wave with an amplitude of 4 mV and a period of 500 μs. The signal was recorded using the system, and the waveform displayed on the App is observed.

A sine wave signal with an amplitude of 4 mV and a period of 500 μs could be identified as shown in Figure 6B. There are, however, glitches and interferences in the waveform, which may be caused by environmental factors, such as the instability of the high-frequency small electrical signal generated by the signal generator, the instability of the power supply, the interference of Bluetooth radio frequency, and the possible power frequency interference. In the subsequent data processing, the transmitted data will undergo filtering and other processing to reduce such interference to a minimum.

#### Stimulation

The circuit is connected as shown in Figure 6C. Given the current-source stimulator, a 10 kΩ load resistance is cascaded into the circuit to convert the current pulse into a voltage pulse. In this test, one channel of the system was used to output biphasic electrical stimulation with different combinations of adjustable amplitude, frequency, and pulse width.

We tested the different conditions of amplitude, frequency, and pulse width. The stimulus pulse frequency displayed by the oscilloscope and the pulse width of a single stimulus waveform is consistent with the set value, and the amplitude is also equal to the product of the set current value and the resistance value of the test resistance. Illustrations of an example output waveform and its zoom-in version are shown in Figure 6D with related adjustable parameters labeled. Real-life stimulus waveform pictures are in the Supplementary section.

### BP-neurogram correlation

In this work, the vagus nerve electroneurogram and blood pressure of rat, which were modulated by periodically injecting phenylephrine, were used to extract CNAPs. Cluster analysis is also conducted to find the feature of CNAP which is most relevant to BP variations. The prototype algorithm has been elaborated in detail in section Signal processing algorithm.

**Figure 7:**
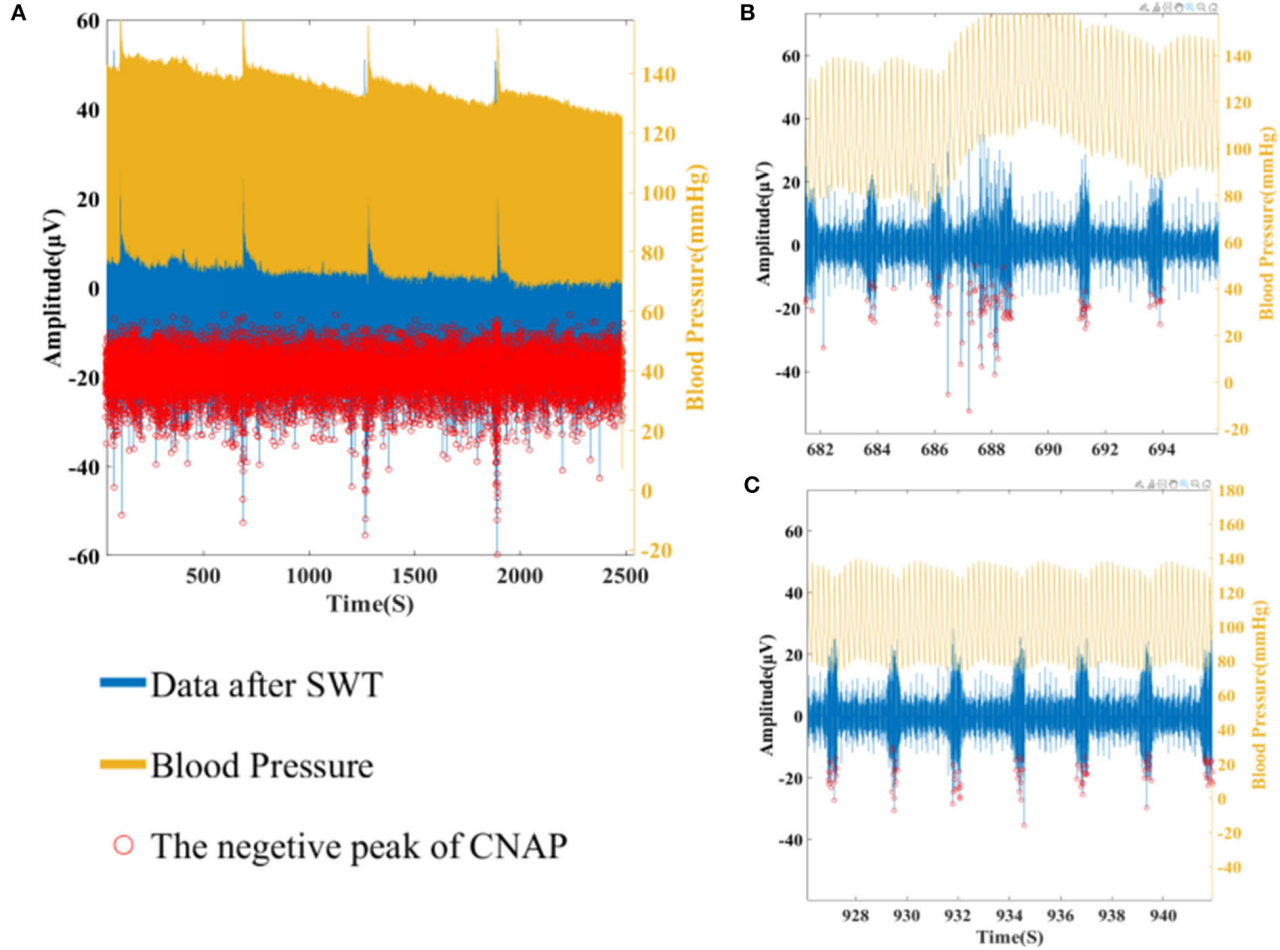
**(A)** Overall CNAP firing and blood pressure changes. **(B)** CNAP firing when blood pressure rises sharply. **(C)** CNAP firing when blood pressure is normal.

**Figure 8:**
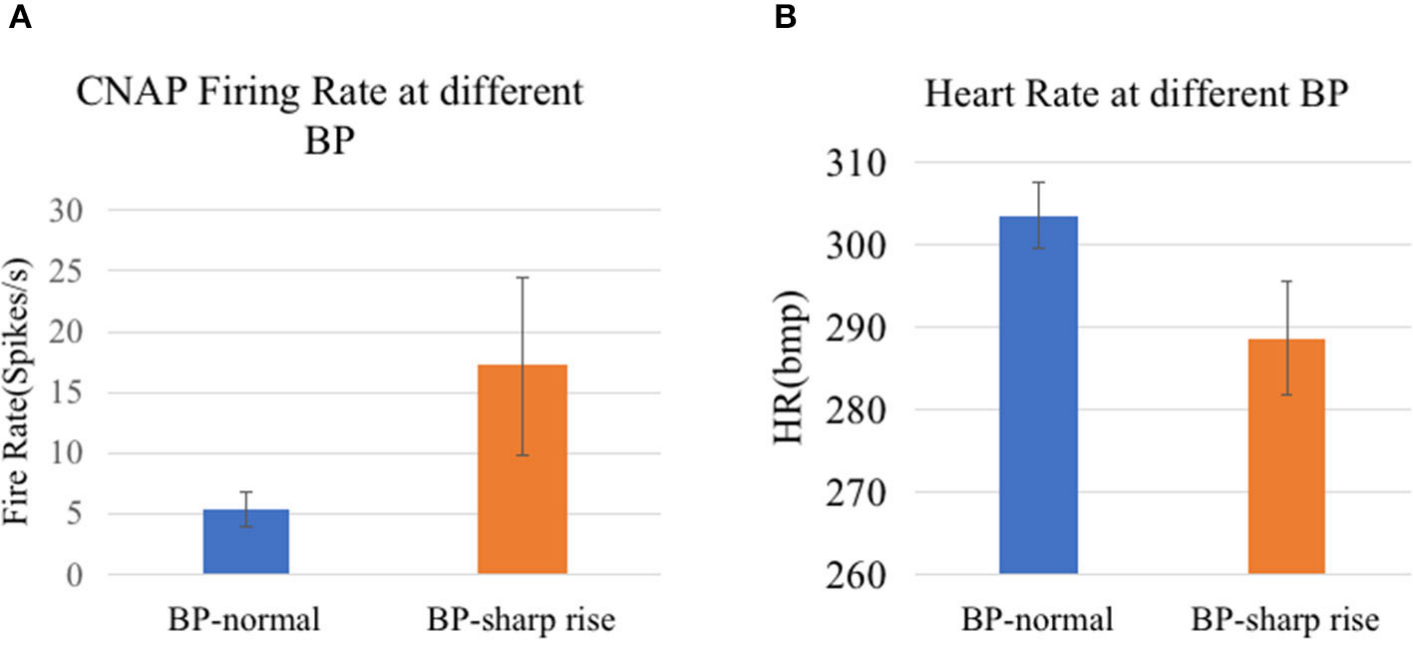
**(A)** CNAP firing rate at different BP (*p* = 0.0023). **(B)** Heart rate at different BP (*p* = 7.4152e-09).

In our analysis, CNAP was found to occur mainly during respiration burst and occasionally during the interval of respiration, which is consistent with the conclusion in other researches. As shown in Figures 7, 8, a marked increase in firing rate of CNAP was observed with the sharp rise in blood pressure. Reflexive reduction in heart rate has also been observed.

In terms of cluster analysis, although different components of CNAPs with different waveforms were successfully separated, there is no clear one-to-one correlation between a specific component and blood pressure value, suggesting that the classification algorithm needs further optimization in the follow-up experiments. Nevertheless, the difference in the overall firing rate does display the potential of this algorithm.

### Vagus nerve stimulation effect in BP regulation

In this study, the blood pressure of healthy rats was used to simulate the blood pressure of patients with RH. The developed system was used to explore the blood pressure-related components of one rat vagus nerve electrical signal through animal experiments and to verify the regulation effect on blood pressure through electrical stimulation.

**Figure 9:**
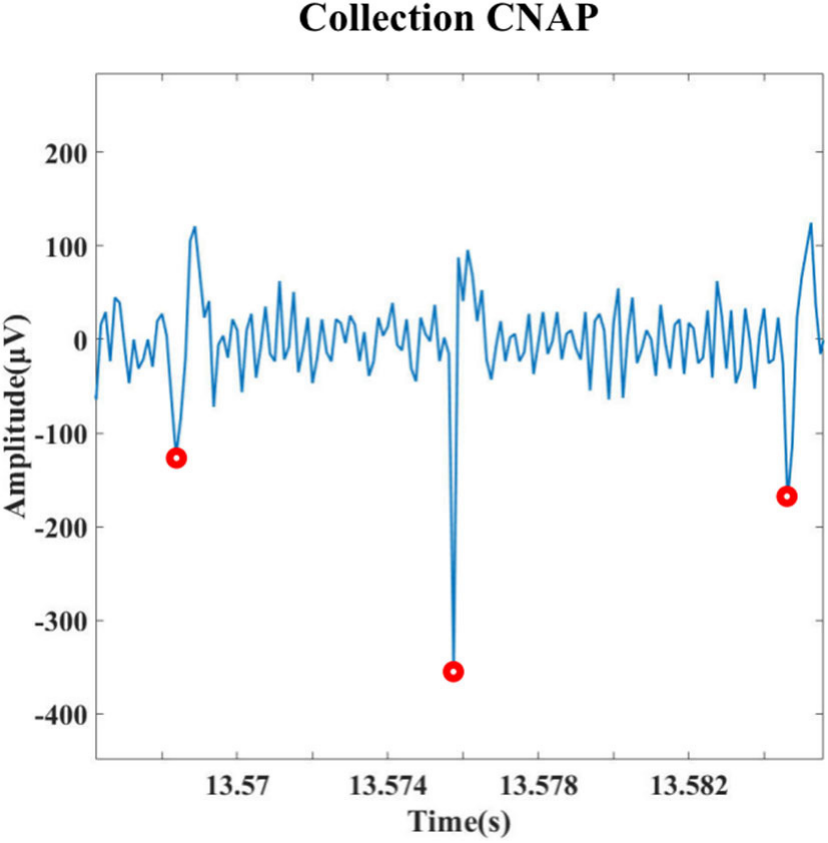
Detection results for spike signal *in vivo* experiments on rats.

Under the condition of an 8 kHz sampling rate, the signals of the left cervical vagus nerve were recorded using the designed system. Figure 9 shows the signal after filtering through a tenth-order Butterworth filter (bandpass is 300–3,000 Hz), the amplitude of the spike is >100 mv, and the signal-to-noise ratio is about 9.93dB, demonstrating that the recording function works well.

**Figure 10:**
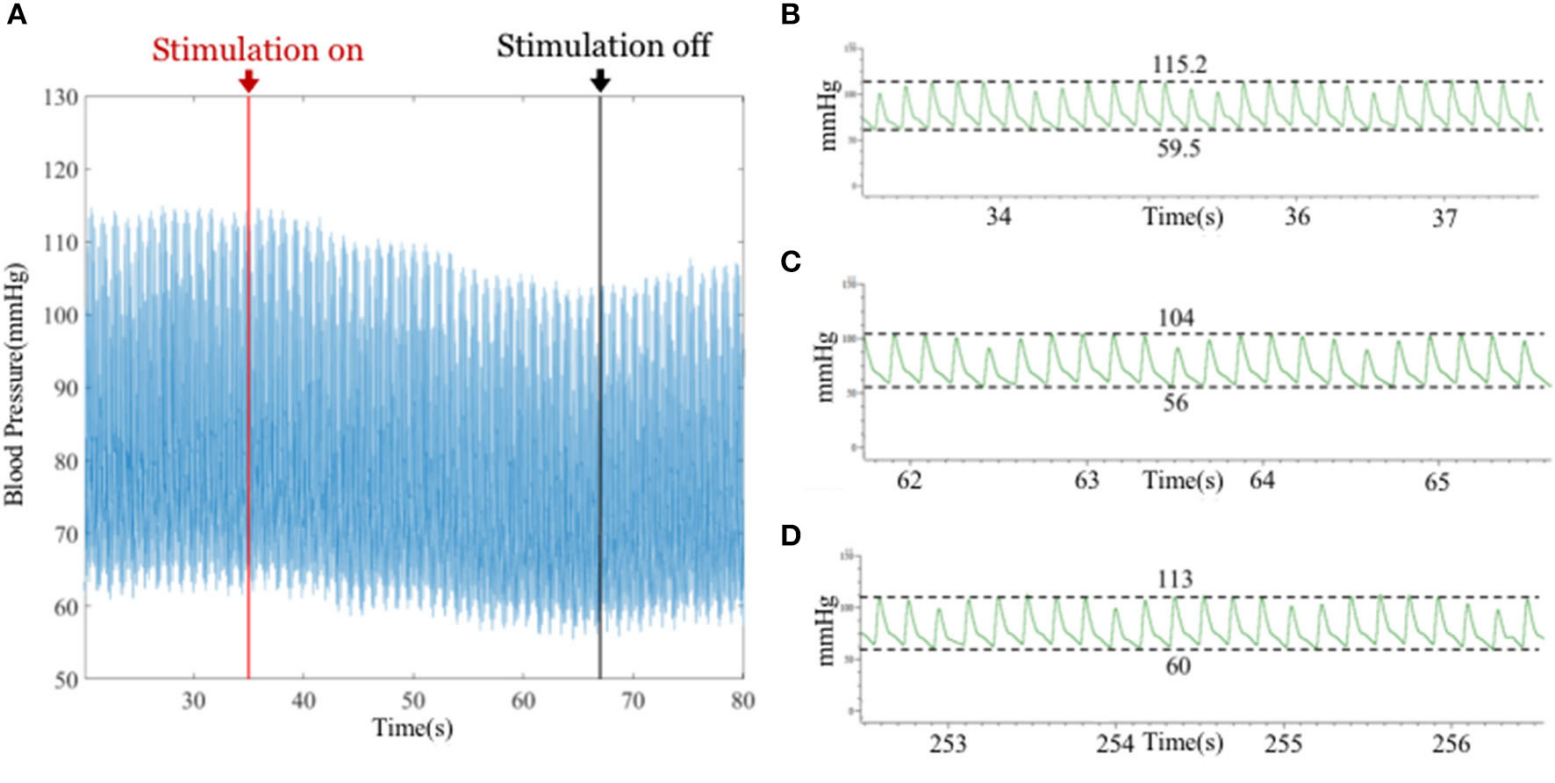
**(A)** Change in blood pressure before and after stimulation. The red arrow indicates the start of stimulation, and the black arrows indicate off-stimulation. **(B)** Blood pressure of rats before electrical stimulation (DBP = 59.5 mmHg, SBP = 115.2 mmHg). **(C)** Blood pressure of rats after the 30 s of electrical stimulation (DBP ≈ 56 mmHg, SBP ≈ 104 mmHg). **(D)** Blood pressure of rats 180s after electrical stimulation stopped (DBP ≈ 60 mmHg, SBP ≈ 113 mmHg).

In the part of blood pressure regulation by electrical stimulation, this experiment verified the antihypertensive effect of electrical stimulation of the vagus nerve by initially trying four sets of parameter combinations. Figure 10 shows the change in blood pressure after stimulation. The magnitude of the stimulation current is 256 μA. It can be observed that the blood pressure decreases continuously after the stimulation function is turned on, and the blood pressure starts to gradually rise after the stimulation function is turned off, which prompted the antihypertension function of vagus nerve stimulation and verified the integrity of the board stimulation function.

## Discussion

In this work, we designed and prototyped a micro-system for electrical signal acquisition and stimulation systems, in the hope to lay an equipment foundation for future closed-loop neuromodulation in hypertension treatment. The key features that we accomplished in this work are as follows: (1) electrical stimulation, the frequency range of which is flexible to either excite or suppress neuron activities; (2) signal recording whose sampling rate is sufficient to intactly collect signals on frequency band we are interested in based on Nyquist law; (3) real-time wireless data communication to provide in-time information about the patients' conditions to make feedback adjustment; (4) preliminary validation on the effect of vagus nerve stimulation to downregulate blood pressure.

Our *in vivo* testing corroborated the idea of modulating hypertension through vagus nerve stimulation using the device developed in this study. Previously, only a few experiments have been conducted on rats using cuff electrodes. We innovated to use the intrafascicular electrodes and further validate the feasibility of this treatment solution.

In terms of the system device, we have realized the basic functions required by a closed-loop system. The present prototype system is capable of recording neural signals (f_s_ = 8 kHz), firing stimulations with flexible parameters (f_stimulate_ <6.7 kHz), and implementing real-time wireless data transmission through BLE. However, there are still a few aspects that need further optimization. Playing a role of a future implantable device, its power consumption, component selection, and packaging should be given more bio-compatible consideration. The battery life per charge could be more enduring by arranging the commands sequences more reasonably—inserting tasks in thread intervals or setting low-power standby mode to cut any possible waste of energy, whereas to fit in the delicate human body, the design of the system could consider using flexible electronics. Deformable substrate would solve the discrepancy between the hardness of rigid implanted devices and soft biological tissues and thus avoid possible immune responses that lead to malfunction of either the device or its surrounding tissues. However, it is not adopted in the current design because of hermetic packaging considerations. Hermetic encapsulation is another critical and essential part of presenting a final clinic product, not only to prevent biochemical reactions between implants and human tissues, but also to deter current leakage and minimize external electromagnetic interference that is likely to mess up with the signals. At present, the mainstream approach is to use a titanium alloy metal shell, leaving little meaning in deciding whether the inside circuit board is soft or not. But in future when means like sealing layer polymer coating have become more mature, soft deformable substrate will be more conducive to wearable applications.

Speaking from the system-designing level, the ultimate goal of this study is to develop a closed-loop neuromodulation device to automatically detect and correspond to abnormal signals by releasing customized electrical stimulation for blood pressure regulation. Such a system comprises two parts: a hardware device that possesses functions of recording and stimulation that make real-time adjustment possible, and a sophisticated algorithm that is able to read blood pressure information from raw neural signals and gives out specific commands on how to perform a proper stimulation intervene. The algorithm part could be further subdivided into two parts: abnormity detection and strategy composition. For abnormity detection, the first step is to interpret blood pressure from neural signals. Preliminary exploration was conducted into looking for underlying patterns of vagus nerve signal in blood pressure modulation. Through signal components, extraction, and cluster analysis algorithms based on machine learning, data sampled by the developed device were analyzed and clustered into seven distinct groups of CNAP. The overall firing rate of CNAP shows a clear correlation with blood pressure, but it cannot be attributable to a classified group. The clustering part of the algorithm needs to be further optimized or replaced with other unsupervised clustering methods.

In regard to the strategy composition part, the appropriate electrical stimulation parameters (stimulating amplitude, frequency, and intervals) for blood pressure regulation should be determined through more animal experiments, both in SD and hypertensive rats or different health conditions or environment settings to generalize the conclusion. Although studies have shown that the stimulating pattern does not make a huge difference in the therapeutic outcome, the optimal stimulation amplitude, frequency, or chronological firing order may vary greatly between individuals in regard to their personal physical differences. It is thus the subsequent pivot to obtain personalized stimulation settings to avoid dogmatical intervening and incorporate them into the system to ensure the best treatment effect. Upon the building and validation of such a closed-loop regulation system, long-term *in vivo* tests are needed to monitor usage and biocompatibility issues.

## Data availability statement

The raw data supporting the conclusions of this article will be made available by the authors, without undue reservation.

## Ethics statement

The animal study was reviewed and approved by Ethics Committee of Human and Animal Experiments, School of Biomedical Engineering, Shanghai Jiao Tong University.

## Author contributions

The manuscript was written by AS and RL, with additional discussions, detailed edits, and feedback provided by XS. AS and JG designed the overall system. RL developed the process and analysis algorithm. AS conducted both *in vivo* and *in vitro* device validation. RL and YL conducted surgical operations on SD rats. AS, RL, and YL recorded and analyzed blood pressure signals with guidance from JW. All authors contributed to the article and approved the submitted version.

## Funding

This project was funded by the National Key R&D Program of China (No. 2022ZD0208601) and the National Natural Foundation of China (No. 62176158).

## Conflict of interest

The authors declare that the research was conducted in the absence of any commercial or financial relationships that could be construed as a potential conflict of interest.

## Publisher's note

All claims expressed in this article are solely those of the authors and do not necessarily represent those of their affiliated organizations, or those of the publisher, the editors and the reviewers. Any product that may be evaluated in this article, or claim that may be made by its manufacturer, is not guaranteed or endorsed by the publisher.
