# Supplementary figures and images for: A system of real-time neural recording and stimulation and its potential application in blood pressure modulation
Source: Front Med Technol. 2022 Aug 10;4:941686. doi: 10.3389/fmedt.2022.941686 (PMC9399767; doi:10.3389/fmedt.2022.941686)

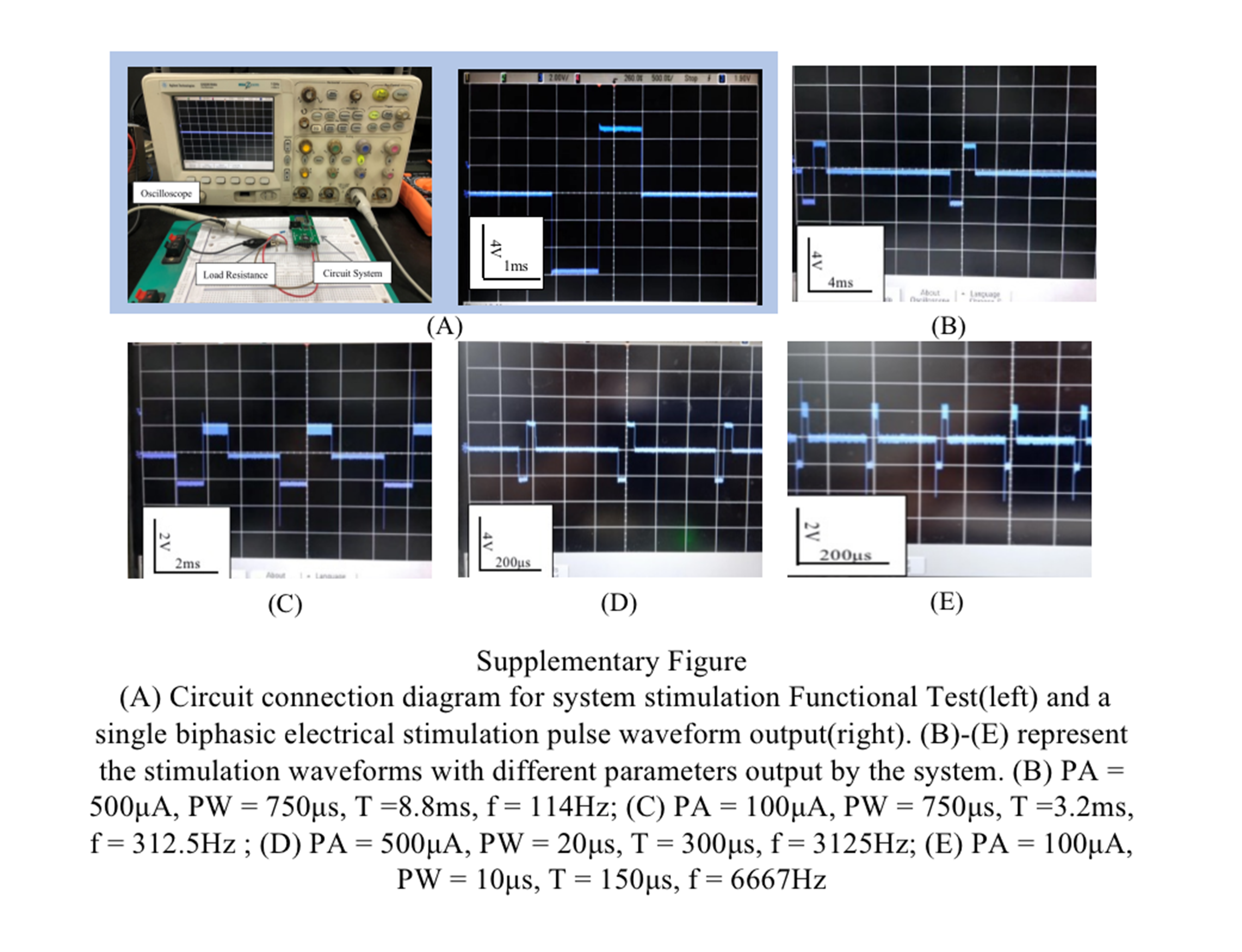

Supplement: Supplementary file 1 [file Image_1.png]
